# Prevalence and associated factors of symptomatic pelvic floor disorders among women living in Debre Tabor Town, Northwest Amhara, Ethiopia

**DOI:** 10.1186/s12905-024-03176-y

**Published:** 2024-06-24

**Authors:** Berihun Assefa Demissie, Merete Kolberg Tennfjord, Tewodros Mihiret, Yohannes Abich, Ashenafi Zemed, Zelalem Mengistu, Solomon Gedlu Nigatu

**Affiliations:** 1 Department of Physiotherapy, School of Medicine, College of Medicine and Health Sciences, Bahir Dar University, Bahir Dar, Ethiopia; 2 School of Health Sciences, Department of Health and Exercise, Kristiania University, Oslo, Norway; 3 Department of Physiotherapy, School of Medicine, College of Medicine and Health Sciences, University of Gondar, Gondar, Ethiopia; 4 Department of Gynecology and Obstetrics, School of Medicine, College of Medicine and Health Sciences, University of Gondar, Gondar, Ethiopia; 5 Department of Epidemiology and Biostatistics, Institute of Public Health, College of Medicine and Health Sciences, University of Gondar, Gondar, Ethiopia

**Keywords:** Pelvic floor disorder, Women, Pelvic organ prolapse, Urinary incontinence, Fecal incontinence, Debre Tabor, Ethiopia

## Abstract

**Background:**

Pelvic floor disorders are a group of disorders affecting the pelvic floor that include clinically definable conditions such as pelvic organ prolapse, urinary incontinence and fecal incontinence. These conditions silently affect millions of women worldwide and related problems are not well disclosed by women due to associated social stigma or lack of access to services in developing countries. Thus, the magnitude and related burden of these conditions vary, and little is known about them. This study was conducted to assess the magnitude and associated factors of symptomatic pelvic floor disorders in Debre Tabor town, Northwest, Ethiopia, from May 30-July 30, 2020.

**Method:**

A community-based cross-sectional study was conducted on child bearing women (> 15 years) who resided in Debre Tabor Town from May 30-July 30, 2020. The participants were selected through multistage systematic random sampling. The data were collected via a structured questionnaire through face-to-face interviews, entered into Epi-info-7.2, and subsequently analyzed using SPSS version 20. The prevalence of pelvic floor disorders was presented along with the 95% CI.

**Results:**

A total of 402 women participated in this study, 59 (14.7%; 95% CI; 11.4, 18.2) of whom reported one or more types of pelvic floor disorders. The most prevalently reported pelvic floor disorders were pelvic organ prolapse (13.9%; 95% CI: 10.9, 17.4), urinary incontinence (10.9%; 95% CI: 7.4, 9.2) and fecal incontinence (7.7%; 95% CI: 5.2, 10.2). Additionally, aging, multiparity and having early marriage (< 18 yrs.) were identified as potential risk factors associated with pelvic floor disorders.

**Conclusions:**

The prevalence of symptomatic pelvic floor disorders in the current study was high. Thus, early detection, preventive and treatment strategies should be considered. In addition, it is better to educate the community and women on the association of early marriage and multiparty with PFDs.

## Background

### Problem statement

Pelvic floor disorders (PFDs) are conditions that affect the pelvic floor muscles and consist of a range of disorders, such as urinary incontinence, pelvic organ prolapse, fecal incontinence and related sensory and sexual dysfunctions [1]. Pelvic organ prolapse, Urinary Incontinence, and Fecal Incontinence are the most common and clinically definable conditions that negatively affect the lives of millions of women globally [2], and the use of Pelvic Floor Disorders will substantially increase, while the need for care will continue to grow through the next 30 years because of population growth and aging [3].

Pelvic floor disorders are becoming major health problems in women worldwide, especially in developing countries. Moreover, these practices silently affect millions of people because access to health care and awareness and autonomy in decision making related to managing Pelvic Floor Disorders are often limited [4, 5]. The magnitude of Pelvic Floor Disorders differs across different countries (11.9%-67.5%) [6, 7]. In high-income countries, especially in the United States, 25% of women report at least one Pelvic Floor Disorder at their lifetime, and Urinary Incontinence is the most common 17.1%, followed by Fecal Incontinence (9.4%) and Pelvic Organ Prolapse (2.9%) [8, 9]. In contrast, the prevalence of Pelvic Floor Disorders in developing countries is greater than that in developed countries; for instance, in Bangladesh, the prevalence of PFDs is 35.3% [10], of which Urinary Incontinence accounts for 23.7%, followed by FI 5.3% and Pelvic Organ Prolapse 16.2% [11]. This finding is supported by a review in developing countries in which the pooled prevalence of Urinary Incontinence was 28.7% (range 5.2–70.8%), that of Pelvic Organ Prolapse was 19.7% (range 3.4–56.4%), and that of Fecal Incontinence was 6.9% (range 5.3–41.0%) [12].

Pelvic floor disorders affect the economic, personal and social aspects of women, especially their daily activities, sexual life, psychosocial wellbeing, quality of life and high cost of health care [13, 14]. The economic impact of Pelvic Floor Disorders in developed countries is high. For instance, in the US population, one in every nine women would undergo surgery for Pelvic Floor Disorder in their lifetime, while approximately 135,000 women would undergo surgery for incontinence and 200,000 for Pelvic Organ Prolapse annually; thus, the lifetime medical cost of a woman with urinary incontinence is 1.8 times greater than that of a woman with no Urinary Incontinence, which was estimated to increase by 45% over the next 30 years [15]. On the other hand, the impact of Pelvic Floor Disorders in developing countries is also high, especially for Urinary incontinence and FI; shame and embarrassment cause women to isolate themselves from friends and family, which increases the economic burden on women, their families and society [16–18]. Similarly, women in Ethiopia are also in a disadvantaged position because Pelvic Floor Disorders are not considered as natural and common while most women don’t take action to improve the situation either due to;related stigma, very low literacy levels, low status of decision making in the households, high workloads even during pregnancy and immediately following childbirth or due to limited access to appropriate services and remain with few options to take appropriate measures [19–21].

There are possible risk factors associated with Pelvic Floor Disorders, of which age is the most frequently identified risk factor [6], while being parous and having multiple parities are among the associated risk factors for having Pelvic Floor Disorders [22]. A heavy load for a longer period of time was also identified as an associated factor of Pelvic Floor Disorders [7, 23]. Obesity (high waist circumference) and overweight (BMI) are among the possible anthropometric factors associated with Pelvic Floor Disorders [24, 25].

Despite family planning, sexual and reproductive health efforts are underway including building a foundation of laws, policies, and programs that support the right to access for services and improve the lives of millions women, large number of women remained with home delivery, high birth and mortality rate in the country [26, 27]. A number of studies about Pelvic Floor Disorders have been performed in Ethiopia, but most have focused only on single symptom of the disorder; pelvic organ prolapse, performed at the facility level [23, 28–31]. There is scant information about Pelvic Floor Disorder in our study area. Therefore, the purpose of this study was to determine the magnitude and identify possible factors influencing PFDs in Debre Tabor town.

## Methods

### Aim

The aim of the study was to determine the magnitude and identify possible factors of symptomatic pelvic floor disorders among women living in Debre Tabor town during the study period.

### Study design and study period

A community-based cross-sectional study was conducted from May–July 2020.

### Study area

The study was conducted on women living in Debre Tabor town, Northwest Amhara, Ethiopia. Debre Tabor is a seat of the South Gondar zonal administration located 667 kms away from Addis Ababa, the capital of Ethiopia. According to the 2010 Central Statistical Agency (CSA) report, the population of Debre Tabor town was estimated to be 78,703 (37,682 males and 41,021 females) [32]. The town is divided into 6 administrative kebeles (the smallest administration unit in Ethiopia). There is one referral hospital and 2 health centers in the town.

### Source population

All women who resided in Debre Tabor town in 2020 (for at least six months) were the source population.

### Study population

All women ≥15 years old and older than three months postpartum lived in Debre Tabor town in 2020.

### Inclusion criteria

Women (≥15 years), lived for >6 months in the town and were present during the data collection period were included.

### Exclusion criteria

Women with Known pregnancy, early postpartum (<3 months), acute stroke (<3 months), speech and hearing impairment, and recent abdominal, urogenital or pelvic surgeries (<6 months) were excluded.

### Sample size determination

A sample size of 414 was calculated by an epidemiologist using a single population proportion formula based on the following assumptions: 95% CI, 20.5% incidence of PFDs [22], 1.5% design effect, 5% margin of error and 10% nonresponse rate.

n = Z^2^P (1-P)/d^2^ = (1.96)^2^ *(0.205) (0.795)/(0.05)^2^ = 251 and using a design effect of 1.5, the sample size was calculated as 251X1.5 = 376; after adding 10% of the nonresponse rate, 376/10 = 38 =  > 376 + 38 = 414, so the final sample size was = 414.

### Sampling technique and procedures

According to the Mayor Office of the town, there are 6 administrative kebeles with an estimated 15,750 households. Using a multistage sampling procedure, three kebeles were selected by the lottery method (50%) (Kebeles 01, 03 and 06). Each kebele had 1500, 2000 and 2500 households, respectively, of which 104, 138 and 172 households were recruited from each kebele, respectively, in proportion. A systematic random sampling method was employed so that the general interval was K = N/n = 6000/414 = 14.49 $\approx$=14 and k was also calculated for each kebele.

Finally, participants were recruited at household level using the household as the study unit; if more than one woman was aged > _15 years, the lottery method was used to include a single woman. The first study participant was selected using the lottery method; then, every 14th household interval was included in this study.

### Dependent variable

Pelvic floor disorders; symptoms of all or either of pelvic organ prolapse, urinary incontinence and fecal incontinence explained by yes/no.

### Independent variables

#### Sociodemographic characteristics: Age, educational status, occupation and age at first marriage and heavy loading activities

Anthropometric factors: Central obesity (WC) and BMI.

Obstetric factors: parity, mode of delivery, number of deliveries, history of abortion and place of delivery.

### Data collection tools and measurements

The data were collected by trained three females, final year undergraduate students of midwifery and nursing, using face-to-face interviews through a home-to-home survey after consent and ascent (in the case of girls < 18 years) were obtained in writing after relevant information was provided. The interview questionnaire was structured and composed of five sections (socio-demographic factors and obstetric history and urinary incontinence, fecal incontinence and prolapse symptoms) which were adapted from an open access valid and reliable short form of the pelvic floor distress inventory (PFDI-20) [33] and recommended by the International Urogynecology Association (IUGA)/International Continence Society (ICS) joint report on the terminology for females in terms of their level of bother [34].

Each PFD domain was assessed based on women’s reporting of symptoms on 3 subscales, the UDI (questions 1–6), the POPDI (questions 7–14) and the CRADI (questions 15–20). Each subscale was dichotomized as Yes/present coded 1 or No/absent coded 0 for each symptom domain to determine the prevalence of PFDs.

Present: if at least one of the questions from any of the pelvic floor disorder categories defines the presence of the problem in that domain, a woman who presents at least one pelvic floor disorder is categorized as a woman with PFD.

Absent: If a women did not report at least one pelvic floor disorder (not present = 0) were categorized as “do not have PFD”.

If symptoms were present, to assess the degree of distress caused by the symptoms, each PFD symptom was assessed by a four-point Likert scale; the individuals were asked “How much are you bothered by the symptoms?”, and the response was rated from ‘not at all = 1, somehow worrisome = 2, moderately worrisome = 3 to ‘quite a bit worrisome = 4’.

The severity index was calculated as the mean value of symptom distress or (number of scores/no. of items) × 25 = (0–100) for each domain: UI/bladder symptoms, FI, and POP. For the severity of PFD, the distress score ranges from 1–100 and is categorized by tertiles (in three parts) “Mild” if score is 3–33, “Moderate” if the score is 34–66 and ‘severe’ if the score is 67–100 [35]. The internal consistency (reliability) of the three subscales was assessed by using Cronbach’s alpha. We found that the UI, FI and POP subscales were 0.74, 0.71 and 0.83, respectively.

Waist circumference: was measured: Align the tape measure at the level of the belly button with the woman having underwear or with light dressing, standing in a relaxed but not contracted abdominal muscle, and breathed out then circle the whole way around the body and back to the starting point [36].

Height: was measured using meter while the woman was standing upright on the level surface.

Weight: was measured using a standard flatness scale and a score of zero while the women were standing and full-bearing with both legs.

### Operational definition

Pelvic Floor Disorders (PFDs): is a group of disorders of lower urinary tract. Participants who had one or more disorders, namely, UI, FI or POP, for 3 months or more.

Urinary incontinence was defined as the complaint of involuntary loss of urine (SUI and UU) for 3 months or more.

Pelvic Organ Prolapse (POP): is a disorder of descent of female organs, including the bladder, small and large bowel, resulting in protrusion of the vagina, uterus or both (3 months and above).

Fecal incontinence was defined as the complaint of involuntary loss of feces for 3 months or more in the form of solid, liquid or passive fecal incontinence, such as soiling without sensation or warning or difficulty wiping clean or coital fecal incontinence.

### Heavy loading activities – Any load > 25 kg that women most frequently carry

Body mass index (BMI): a biomarker index calculated by dividing weight in kg and height in meters squared (kg/m^2^) and was obtained based on age- and sex-specific BMI information from the WHO [37].

Central obesity (WC): defined as an excess accumulation of fat in the abdominal area, particularly due to excess visceral fat, measured as waist circumference (WC < 79 cm = ideal, 80–88 cm = high and WC > 89 cm = very high) [38].

### Data quality control

The original questionnaire was adapted from the PFDI-20, was prepared in the English language and was subsequently translated into Amharic (a local language). Forward translation was performed by two bilingual translators for the applicability, simplicity, and understandability of the tool. The first translation was performed by the primary investigator and an English language instructor independently. Then, auditing and crosschecking were performed between the translators. And finally with modifications, the questionnaire was adapted and used. A pretest was performed and modifications were made; additional nonverbal explanations for some terms, e.g., Vagina, POP and gas were provided. The data collectors were trained for one day and supervised during the data collection. The collected data were checked for completeness, accuracy and clarity by the primary investigator during the data collection period.

### Data processing and analysis

The collected data were checked for completeness and clarity; coded and entered into Epi Info 7.2; cleared and analyzed by using SPSS version 25. Descriptive (frequency, mean, median and cross tabulation), bivariate and multivariable analyses were performed. Model fitness was checked with the Hosmer and Lemeshow test, and the *p* value was 0.23. Factors with a *p* value < 0.2% in the bivariate logistic regression analysis were analyzed using multivariable logistic regression to control for possible confounders and examine the association between the dependent variable and different independent variables. The AORs were considered significant at a *p* value < 0.05 and were used for discussion.

## Results

### Sociodemographic characteristics

Out of four hundred fourteen women, 402 participated in this study, for a response rate of 97.1%. The median age of the participants was 32 (IQR 25–45). Approximately 320 (79.6%) participants were of reproductive age (15–49 years). The majority (88.3%) were also Orthodox Christians. More than half (54%) of the participants were married, and 72 (17.9%) were divorced/separated/widowed.

Among the married participants, approximately 125 (43.3%) were engaged while they were under 18 years old. Body mass index (BMI) was calculated for all women, 36 (9%) were underweight and 8 (2%) were overweight. Approximately 29 (7.2%) women had very high central obesity, as measured by large waist circumference.

As shown in Table 1, regarding the age distribution, the prevalence of PFDs was 39% in women aged ≥ 50 years and 17.2% in women aged ≤ 49 years. On the other hand, the distribution of PFDs was greater (37.5%) among overweight women (BMI > 25).

**Table 1:** Socio-demographic characteristics and cross tabulation with PFDs, Debre Tabor, 2020

Characteristics	Category	Frequency		PFDs	
		N	%	N	%
**Age**	< 18 19–34 35–49 > 50	13 208 99 82	3.2 51.7 24.6 20.4	1 9 17 32	7.7 4.3 17.2 39.0
**Age of 1st marriage (*n* = 289)**	< 18 > 18	125 164	43.2 56.8	36 20	28.8 12.2
**Educational status**	Non educated Primary 2ndry ≥ College	65 89 137 111	16.2 22.1 34.1 27.6	22 19 8 10	33.8 21.3 5.8 9.0
**Occupation**	Student housewife merchant daily laborer permanent	80 138 62 47 75	19.9 34.3 15.4 11.7 18.7	3 37 9 4 6	3.8 26.8 14.5 8.5 8.0
**Exposure of heavy loading**	No Yes	258 144	64.2 35.8	29 30	11.2 20.8
**BMI**	Underweight Normal weight Over weight	36 358 8	9.0 89 2.0	2 54 3	5.6 15.1 37.5
**WC**	Ideal High Very high	232 141 29	57.7 35.1 7.2	17 26 16	7.3 18.4 55.2

### Obstetric characteristics

Approximately 249 (61.9%) of the women were parous, and 205 (51%) were multiparous, with a mean of 2.89 children. Among the women who delivered, one hundred sixty-four (65.9%) had institutional deliveries, eighty-three (20.6%) of whom had 4–6 births. The majority of respondents had their first delivery vaginally (91.2%), and sixty-nine (27.7%) women had their first delivery before the age of 18 years. Regarding parity, the distribution of the prevalence of PFDs among parous women was greater (21.7%) than that among nulliparous women (3.3%).

### Prevalence of PFDS

**Fig. 1:**
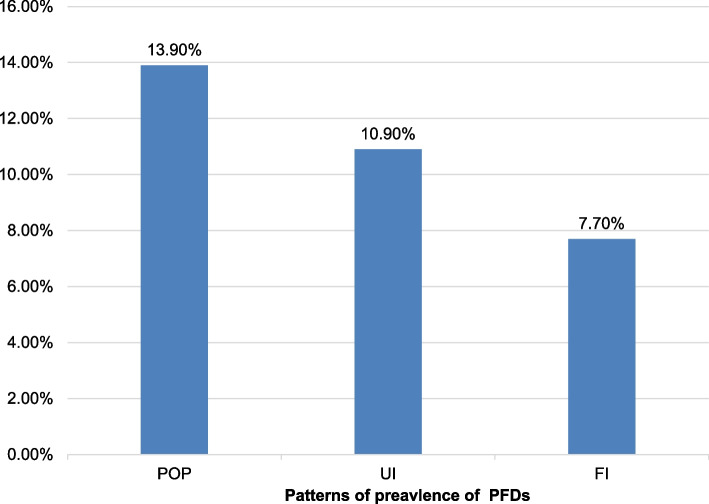
Prevalence of pelvic floor disorders among women living in Debre Tabor, Ethiopia, 2020

The overall prevalence of symptomatic PFDs in this study was 14.7%, 95% CI = 11.4%-18.2%, and POP was the most prevalent domain of PFDs (13.9%, 95% CI = 10.9%-17.4%), followed by UIs (10.9%, 95% CI = 8.2%-13.9%) and FIs (7.7%, 95% CI = 5.2%-10.2%) as shown below in Fig. 1.

Among women who reported symptoms of PFD, 10.7% of women with POP and 9.1% of women with UI reported moderate distress symptoms, while all women with FI reported mild symptoms.

(Severity index = (total scores of each domain) × 25/ (No of items) =  > (3–33.3-mild, 34–66 = moderate, 67–100% = severe); no severe symptoms in any of the PFD domains were reported.

### Factors associated with the incidence of pelvic floor disorders

Binary logistic regression analysis was conducted for each predictor variable, and PFD was shown to be associated with age, parity, multiparity, vaginal delivery, BMI, central obesity (WC), early first marriage, abortion history, occupation, home delivery, exposure to heavy loading activities and low educational status.

After multivariable logistic regression analysis was conducted, older patients (aged > 49 yrs.), multiparity (> 3) and early marriage (< 18 yrs.) was associated with PFDs.

Women older than 49 years were more likely to develop PFDs (odds ratio (AOR) of 4.93 (95% CI = 1.09–22.35)) than younger women were. Women with multiple (> 3) deliveries were 2.27 times more likely to develop PFDs (AOR, 95% CI, 1.02–4.88) than those with (≤ 3) deliveries. As shown in Table 2, women who were in early marriage (< 18 yrs.) were 2.67 times more likely to develop PFD (AOR, 95% CI 1.23–5.80) than older adults (> 18Yrs.)

**Table 2:** Factors and association with prevalence of PFDs among women, Debre Tabor, 2020

Predictor variable		COR (95% CI)	AOR (95% CI)	*P*-value
Parity (*n* = 249)	≤ 3 ≥ 4	1 4.11(2.19–7.71)^a^	1 2.27(1.02–4.88)^a^	0.000
Age (*n* = 402)	15–32 33–49 > 50	1 3.38(1.49–7.66)^a^ 12.48(5.75–27.09)^a^	1 2.047(.57–7.38) 4.93(1.09–22.35)^a^	.274 .039
Heavy loading activities (*n* = 249)	Yes No	2.07(1.19–3.62)^a^ 1	2.08(0.96–4.53) 1	.064
Educational status (*n* = 402)	Non educated primary 2ndry College & above	5.167(2.25–11.83)^a^ 2.741(1.202–6.25)^a^ 0.626(0.239–1.64) 1	2.16(0.64–7.24) 2.16(0.71–6.51) 0.77(0.22–2.67) 1	.211 .172 0.68
Age of marriage(*n* = 289)	< 18 > 18	2.91(1.59–5.34)^a^ 1	2.67(1.23–5.80)^a^ 1	.013
Place of delivery(*n* = 249)	Home Institution	1.94(1.05–3.59)^a^ 1	0.27(0.1–0.73) 1	.10
Mode of delivery(*n* = 249)	Vaginal Cesarean	6.39(0.84–48.68) 1	6.67(0.73–61.30) 1	.094
WC(*n* = 402)	< 79 80–88 > 89	1 2.86(1.49–5.49)^a^ 15.56(6.44–37.63)^a^	1 0.46(0.16–1.37) 2.18(0.55–8.68)	.164 .268
Abortion(*n* = 249)	No Yes	1 2.49(1.13–5.48)^a^	1 2.24(0.69–7.30)	.181

^a^assigned for variables significantly (*P* < 0.05) associated with the outcome (1 = reference)

## Discussion

This cross-sectional community-based study was conducted among women aged > 15 years in Debre Tabor town, Ethiopia, using a standardized self-reported pelvic floor distress inventory (PFDI-20) tool. Hence, the aim of the present study was to determine the magnitude and associated factors of PFDs among women in Debre Tabor, and the prevalence rate of PFDs was 14.7%. This result is greater than that of a previous study conducted in Dabat, Ethiopia (11.9%) [7], and lower than that of Kersa, Ethiopia (20.05%) [39], studies in USA (25%) [9], Bangladesh (35.5%) [11] and Turkey (67.5%) [6]. This variability might be attributed by differences in participants, for example, in the study conducted in USA, majority (87.2%) of women were parous [9] while (61.9%) in the current study whereas in a study in Turkey, majority of women (75%) were parous [6] and their mean age was greater (mean age 40.76 ± 12.6 years, 15–86 years) than women in the current study (median age 32.15–67 years).

On the other hand, in the Bangladesh National Population-based Study (64.3%) women had, more than 3 deliveries [11], while fewer (34.8%) had deliveries in the current study. Similarly, a previous cross-sectional community-based study in Ethiopia involving ever-married women revealed a mean age of 36.5 ± 13.15–80 years and a high mean number of deliveries of 5.6 (1–16) [39], while the current study included 28.1% of single women with a median age of 32 (15–67 years) years and a mean number of deliveries of 2.9 (0–6).

In this study, POP was the most frequently reported PFD (13.9%), followed by UI (10.9%) and FI (7.7%). The prevalence of POP was consistent with that of reported in a previous study conducted in Bench Maji, Ethiopia (13.3%) [23], and was higher than that of reported in previous studies in Dabat and other countries [7, 40, 41]. Whereas it was lower than that of reported in other recent community-based studies in Ethiopia and Tanzania [30, 42], although it fell within the range (3.4–56.4%) of prevalence rates worldwide [12]. The prevalence of UI is also higher than that reported in previous studies in Ghana (5.2%) [43] and lower than that reported in other studies [16, 28, 44–46], while it falls within the range (5.2%-70.8%) of previous reports [12]. On the other hand, the prevalence of FI is consistent with that reported in a study conducted on racially diverse women (6%) [47] but less than that reported in a population survey in the U.S. (14.4%) [48]. This difference may be explained by a greater number of deliveries, heavier physical workload [7], engagement in manual work even while pregnant or shortly after delivery, and poor nutritional status [12] among Ethiopian women.

The pattern of PFD might vary in relation to the exposure of women to contributing and other socio-demographic factors. In this study, POP was the most prevalent PFD, which is different from the findings of Asian and African studies in which UI was the most commonly reported POP [45, 49, 50], while it was the least commonly reported PFD next to FI and UI in Western countries [9, 51]. This difference may be because women in Ethiopia are more likely to experience frequent vaginal deliveries and multiparty than women in Western countries are. For instance, the prevalence rates of UI and FI may vary with biological or racial differences, and a lower incidence of UI has been observed among black women than among white women [3]. The difference in pattern could be explained by the greater urethral closure pressure in black women during maximum pelvic muscle contraction than in other races [52].

This study showed that multiparity (> 3 children) was significantly (*P* < 0.05) associated with PFD, with an odds ratio of 2.27; this finding is supported by findings from other studies [6]. This may be because multiparity is one of the potential factors of levatorani muscle avulsion, and over distention, muscle weakness and increased hiatal area are prime risk factors for POP [53]. Age (> 35 years) was also significantly associated with PFDs, and the association increased as age increased (50 years), with an odds of 4.93 years, which was supported by findings from other studies [22]. For instance, aging has muscular, hormonal, and neurological effects on the pelvic floor, especially in parous women, and may cause pelvic floor weakness and dysfunction [54]. Similarly, early marriage (< 18 yrs.) was associated with PFDs, with an odds of 2.67 times greater than that associated with engaging later. This may be because girls who marry early may start early sexual intercourse, which may affect pelvic floor structures, and may also have many child bearings. In addition, young mothers have less influence and less control, leading to decision-making about their nutritional status, health care and household management [55].

Services, related to early identification and management of PFDs remained with huge gaps; which is influenced by distance to access, economic and level of health literacy and COVID_19 pandemic, hence strategies and opportunities such as telemedicine should be implemented to assure equity and quality of health sevices to PFDs [56]. Along with the routine Pelvic Floor exercises, the recently advanced treatment options of using artificial anal sphincter implantation that improved the external anal sphincter muscle tension with a positive correlation between its increase and the clinical outcome for patients with fecal incontinence [57] and hemorrhoidal disease is recommended to integrate with [58].

### Limitations of the study

Because the patients were self-reported, they might have been missed due to recall bias. There would be socially desirable bias, even if we used female data collectors to minimize it. Thus, the prevalence of PFDs reported in this study might underestimate the true magnitude of PFDs.

## Conclusion

In this study, the prevalence of PFDs was high, and this magnitude shows that PFDs are public health problems for women in the study area. Age, multiparity and early marriage were identified as potential factors associated with PFDs. 

For policy makers and women’s health promoters, this is the time to underline PFDs as women’s health problem and initiate preventive and treatment strategies. Particularly, for the Ethiopian Ministry of Health particularly; Maternal and Child Health Directorate, Amhara Regional health Bureau and Debre Tabor Town health department, awareness raising, early screening and treatment services and should be incorporated into family planning, sexual and reproductive health programs to reduce the risk of having PFDs due to multiparty (> 3 children) and early marriage (< 18 yrs.) and physiotherapy departments of the University universities also to include services for PFDs in their clinical practices. For researchers, taking this result as an eye opening baseline, we encourage to conduct new studies using high-quality study designs.

## Data Availability

All essential datasets generated and/or analyzed during the current study are not publicly available due to privacy issues but are available from the primary author (Berihun Assefa).

## Abbreviations

BSc: Bachelor of Science
CRADI: Colorectal Anal Distress Inventory
FI: Fecal Incontinence
Hrs: Hours
ICIQ-VS: International Consultation on Incontinence Questionnaire Vaginal Symptoms and ICI- International Consultation on Incontinence
MPH: Masters of Public Health
MPT: Masters of Physiotherapy
MSc: Master of Science
OAB: Overactive Bladder
OBGY: Obstetrics and Gynecology
OPD: Outpatient Department
OR: Odds ratio
PFDI: Pelvic Floor Distress Inventory
PFDs: Pelvic Floor Disorders
POPCI: Pelvic Organ Prolapse Distress Inventory
SPSS: Statistical Package for the Social Sciences
UI: Urinary Incontinence
WC: Waist circumference
